# Role of Lung Function Genes in the Development of Asthma

**DOI:** 10.1371/journal.pone.0145832

**Published:** 2016-01-11

**Authors:** Hideyasu Yamada, Hironori Masuko, Yohei Yatagai, Tohru Sakamoto, Yoshiko Kaneko, Hiroaki Iijima, Takashi Naito, Emiko Noguchi, Satoshi Konno, Masaharu Nishimura, Tomomitsu Hirota, Mayumi Tamari, Nobuyuki Hizawa

**Affiliations:** 1 Department of Pulmonology, Graduate School of Comprehensive Human Sciences, University of Tsukuba, Tsukuba, Ibaraki, Japan; 2 Tsukuba Medical Center, Tsukuba, Ibaraki, Japan; 3 Department of Medical Genetics, Graduate School of Comprehensive Human Sciences, University of Tsukuba, Tsukuba, Ibaraki, Japan; 4 Laboratory for Respiratory Diseases, Center for Genomic Medicine, Institute of Physical and Chemical Research, Tsurumi, Kanagawa, Japan; 5 First Department of Medicine, School of Medicine, Hokkaido University, Sapporo, Hokkaido, Japan; Zhongshan Hospital Fudan University, CHINA

## Abstract

Although our previous GWAS failed to identify SNPs associated with pulmonary function at the level of genomewide significance, it did show that the heritability for FEV_1_/FVC was 41.6% in a Japanese population, suggesting that the heritability of pulmonary function traits can be explained by the additive effects of multiple common SNPs. In addition, our previous study indicated that pulmonary function genes identified in previous GWASs in non-Japanese populations accounted for 4.3% to 12.0% of the entire estimated heritability of FEV_1_/FVC in a Japanese population. Therefore, given that many loci with individual weak effects may contribute to asthma risk, in this study, we created a quantitative score of genetic load based on 16 SNPs implicated in lower lung function in both Japanese and non-Japanese populations. This genetic risk score (GRS) for lower FEV_1_/FVC was consistently associated with the onset of asthma (*P* = 9.6 × 10^−4^) in 2 independent Japanese populations as well as with the onset of COPD (*P* = 0.042). Clustering of asthma patients based on GRS levels indicated that an increased GRS may be responsible for the development of a particular phenotype of asthma characterized by early onset, atopy, and severer airflow obstruction.

## Introduction

Reduced lung function at birth is a risk factor for asthma at 10 years of age [1]. Recently, genes implicated in fetal lung development have also been reported to influence asthma susceptibility and treatment response, supporting the involvement of deregulated lung development in the early ontogeny of asthma [2]. Adverse factors affecting lung development during fetal life and early childhood reduce the attainment of maximum lung function and accelerate lung function decline in adulthood, thereby predisposing individuals to reduced lung function and increased respiratory morbidity, particularly asthma and COPD, throughout life [3]. We have previously reported that *TSLP* gene variants, as a potential susceptibility locus to asthma [4], was associated with lower lung function in healthy individuals, which is also in line with the contention that genetic determinants of lung function influence susceptibility to asthma [5]. In addition, several cluster analyses of asthmatic patients including ours indicated that lung function was among the important discriminating factors of the asthma phenotype [6, 7]

To date, genomewide association studies (GWASs) on pulmonary function including predicted percentage of forced expiratory volume in 1 second (%FEV_1_) and ratio of forced expiratory volume in 1 second to forced vital capacity (FEV_1_/FVC) have identified a number of risk loci in multiethnic populations [8–13]. Although our previous GWAS failed to identify SNPs associated with pulmonary function at the level of genomewide significance in a Japanese population, it did demonstrate that the heritability of pulmonary function can be explained by additive effects of multiple common SNPs, providing compelling evidence for a strong genetic influence on FEV_1_/FVC [14]. Furthermore, our study indicated that lung function genes identified in previous GWASs in non-Japanese populations accounted for 4.3% to 12.0% of the entire estimated heritability of FEV_1_/FVC in a Japanese population. Therefore, in the current study, we constructed a multi-SNP genetic risk score (GRS) for reduced FEV_1_/FVC using genotype information for 16 genes associated with lower FEV_1_/FVC in a GWAS of Japanese populations as well as in previous GWASs of non-Japanese populations. In this study, we investigated the relationship between the onset of asthma and genetic susceptibility to lower lung function as indicated by a multi-SNP GRS, and also attempted to identify a relevant asthma phenotype associated with an increased multi-SNP genetic score.

## Materials and Methods

### Ethics Statement

This study was approved by the Human Genome Analysis and Epidemiology Research Ethics Committee of the University of Tsukuba and by the Human Genome/Gene Analysis Research Ethics Review Committees of the Tsukuba Medical Center, RIKEN, and the Hokkaido University School of Medicine. Written informed consent was obtained from each participant in accordance with institutional requirements and the principles of the Declaration of Helsinki (IRB approval date: January 31st, 2008; IRB number: 2008-01-31).

### Study populations

We defined 3 cohorts. (1) “Tsukuba cohort”: 1364 healthy adults who visited the Tsukuba Medical Center for an annual health checkup and 578 asthmatic patients from the Tsukuba University Hospital and its affiliated hospitals [5, 6, 15]. For the Tsukuba cohort, we conducted GWAS genotyping in 967 healthy adults and 244 asthmatic patients [14, 15]. (2) “Hokkaido cohort”: 998 healthy adults and 565 asthmatic patients from the Hokkaido University Hospital and its affiliated hospitals [15, 16]. (3) “COPD cohort”: 562 COPD patients were studied. Of those, 307 patients were from the Hokkaido University Hospital and its affiliated hospitals [17]; the other 255 patients were newly recruited at the University of Tsukuba and its affiliated hospitals. The participants’ characteristics are summarized in S1 Table. The data were presented as means ± SDs unless otherwise indicated.

### Diagnosis criteria for asthma and COPD

Asthma was diagnosed by respiratory physicians according to the American Thoracic Society criteria [15]. COPD patients were enrolled when they showed chronic irreversible airflow limitation defined by an FEV_1_/FVC < 70% after the inhalation of a β2-agonist. Patients with COPD were carefully screened to exclude those with asthma [17]. Patients were also excluded if they had other significant respiratory diseases, such as lung cancer, eosinophilic pneumonia, or pulmonary tuberculosis, on the basis of the questionnaire and chest X-ray results [5, 14, 15]

### Pulmonary function data

Spirometry was required in order to meet the criteria established by the Japanese Respiratory Society (JRS) [18]. For the 1364 healthy adults who visited the Tsukuba Medical Center for an annual health checkup, pulmonary function was measured using an electronic spirometer (Autospiro series; Minato Medical Science, Osaka, Japan), as described previously [14, 19]. Briefly, well-trained spirometry technicians continuously monitored the procedure and reviewed the flow-volume curves to ensure adherence to the standards. Participants performed up to 3 forced expiratory maneuvers so that acceptable maneuvers could be obtained. Bronchodilation was not applied. Annual decline of FEV_1_ was calculated as previously described [19]. Data for FEV_1_/FVC were available for all the participants, and data for annual decline of FEV_1_ were available for 803 nonasthmatic, non-COPD individuals [19].

### Genotyping

Two hundred forty-four patients with asthma and 967 nonasthmatic, non-COPD controls underwent GWAS genotyping [14, 15]. For those individuals without GWAS genotyping (899 asthmatic patients, 562 COPD patients, and 1395 nonasthmatic, non-COPD healthy individuals), genotypes for 16 SNPs were defined using the TaqMan allele-specific amplification method (Applied Biosystems, Foster City, CA, USA), as described previously [4].

### Statistical analysis

Considering the presence of allelic heterogeneity and different linkage disequilibrium patterns according to different ethnicities, 24 genes previously associated with FEV_1_/FVC with probability values less than 5.0 x 10^−8^ (S2 Table) were tested for gene-level replications rather than for SNP-level replications in Japanese nonasthmatic, non-COPD individuals (N = 967) in the Tsukuba cohort [14]. We selected a top SNP with the strongest statistical evidence of association in each region extending ± 100 kb from each candidate gene for lung function, as described previously [14]. Because 16 of the 24 genes were nominally associated with FEV_1_/FVC in the Japanese nonasthmatic, non-COPD individuals, we used the genotype information from these 16 genes to calculate the multi-SNP genetic risk score (GRS) for lower FEV_1_/FVC in each participant. The GRS, which combines the modest effects of multiple SNPs into a single variable, is calculated as the weighted sum of the number of high-risk alleles, where the GRS for person is

$$\text{GRS}i=\overset{16}{\underset{\text{k}=1}{\sum }}\left|\beta_{k}\right|RA_{k}$$

GRS*i*: GRS for individual *i*

β*k*: regression coefficient of SNP*k* as the weight of each risk allele derived from our GWAS for FEV_1_/FVC

RA*k*: number of risk alleles for SNP*k* (0, 1, or 2)

The score was then divided by the highest risk score multiplied by 100 to rescale the scores to a range between 0 and 100. We used a linear mixed model (SPSS version 22) to compare the GRSs of healthy participants and of patients with asthma or COPD. The standardized coefficient “β” was selected as the weight (instead of, for example, simply counting the number of risk alleles) because it considers both the expected magnitude and direction of effect of an individual SNP.

To estimate the dose effect of a GRS for asthma, using 1143 patients with asthma and 2362 healthy adults, we conducted a Cochran-Armitage trend test for the prevalence of asthmatic patients according to the different ranges of the GRS using R software (version 3.1.1) [20].

To examine whether the significant association between a GRS and asthma is driven by a single SNP, the association analysis was repeated by recalculating the GRS after excluding the genotype information from each of the 16 SNPs (a leave-one-out sensitivity analysis).

To identify distinctive patient characteristics related to an increased GRS level, we applied k-means cluster analysis to the asthmatic patients by including the GRS as a discriminating variable. For this cluster analysis, among a total of 1143 asthma patients in the study, 1063 patients with complete data on GRS, age at onset, and %FEV_1_ were studied. Because several cluster analyses of asthma including ours showed that age at onset of asthma and %FEV_1_ were the most important discriminators for asthma [6], in addition to the GRS, age at onset of asthma and %FEV_1_ were intentionally selected for this cluster analysis. Differences in demographic and clinical characteristics between the resulting clusters were analyzed using ANOVA for continuous normally distributed variables, chi-squared tests for categorical variables, and the Kruskal-Wallis test for non-normally distributed variables. Although our previous cluster analysis identified 6 clusters largely based on age at onset of asthma and %FEV_1_ [6], in the current study, the clustering that resulted in 5 groups was chosen for further analysis because of a clinical judgment that going from 5 to 6 groups resulted in patient clinical characteristics becoming more homogeneous rather than more distinct; especially, the number of clusters (*k*) that gave the maximum difference in the GRS score among the *k* clusters was 5 (S3 Fig). Then, given the 5 distinct clusters, we applied multinomial logistic regression analysis to examine association of a GRS with each of the 5 asthma clusters, which was adjusted for sex, age, atopy, smoking index group, and the cohorts.

To explore the biologic pathways related to a GRS, the Gene Relationships Among Implicated Loci (GRAIL) program was applied [21]. Briefly, GRAIL is a text mining tool that identifies nonrandom, evidence-based links between genes using PubMed abstracts. On the basis of the textual relationships between genes, GRAIL assigns a probability value to each region suggesting its degree of functional connectivity among genes at different loci. As input, we uploaded 16 SNPs used for the construction of a GRS. The GRAIL algorithm is available online at https://www.broadinstitute.org/mpg/grail/. We used the VIZ-GRAIL software (http://www.broadinstitute.org/mpg/grail/vizgrail.html) [22], which was implemented as 2 separate perl scripts, to visualize the results from the GRAIL analysis with default parameters. To explore the biologic relevance of a GRS, we also conducted pathway enrichment analysis for FEV_1_/FVC using MetaCore^™^ software (Thomson Reuters, Philadelphia, PA, USA), an integrated software suite for functional analysis of all omics data, which connects the inputted SNPs to biologic effect or function to define the biologic relevance of the SNPs to lower lung function. We uploaded SNPs with probability values less than 0.05 in our GWAS of FEV_1_/FVC (n = 24,309) for the enrichment analysis, and we then compared the results of GRAIL and MetaCore^™^ to identify possible clues to the molecular events leading to asthma.

## Results

Among the 24 genes associated with FEV_1_/FVC in 6 previous GWASs in non-Japanese populations, we found that 16 genes were nominally associated with FEV_1_/FVC in a Japanese nonasthmatic, non-COPD population (S3 Table). A multi-SNP genetic risk score, or GRS, calculated on the basis of these 16 SNPs for healthy individuals in the Tsukuba cohort follows a normal distribution (S1 Fig). The GRS for lower FEV_1_/FVC was significantly correlated with FEV_1_/FVC in 1364 healthy individuals in the Tsukuba cohort (*R*^*2*^ = 0.040, *P* = 4.1 × 10^−14^), but not with an annual decline in FEV_1_ in 803 healthy individuals (*R*^*2*^ = 0.0001, *P* = 0.34) (S2 Fig).

Compared with GRS levels in healthy individuals, we found significantly higher levels of GRS in patients with asthma in the Tsukuba cohort (*P* value = 0.033) (Table 1). We replicated this significant association between a GRS and asthma in the independent Hokkaido cohort (*P* value = 0.014). GRS levels were consistently higher in patients with asthma than in those in healthy adults; in the combined analysis of the Tsukuba and Hokkaido cohorts, higher levels of GRS were associated with the presence of asthma with the adjusted *P* value of 9.6 × 10^−4^. These results did not change when individuals with a smoking history of over 10 pack-years were excluded from the analysis. In addition, GRS levels were also significantly higher in patients with COPD (Table 1).

**Table 1:** Association between genetic risk score and onset of asthma or COPD.

cohort	Tsukuba cohort		Hokkaido cohort		Combined		Combined (smoking index ≦ 200)		COPD cohort	
status	HV	BA	HV	BA	HV	BA	HV	BA	HV	COPD
Number	1364	578	998	565	2362	1143	1604	857	2362	562
GRS (mean)	56.06	56.79	56.09	56.96	56.07	56.87	55.97	56.73	56.07	56.53
GRSh (SD)	7.2	6.7	7.6	7.3	7.4	7.0	7.3	7.1	7.4	6.8
*P* value	0.037		0.014^b^		0.0022		0.013		0.021^b^	
*P* value (corrected)^a^	0.033		0.007^b^		9.6×10^−4^		0.0020		-	

HV, healthy volunteer; BA, bronchial asthma; COPD, chronic obstructive pulmonary disease; SD, standard deviation

^a^Corrected for sex, age, smoking index group, and atopy. In the combined analysis, cohort effects was also adjusted using the random effect model.

^b^one-sided test

**Fig 1:**
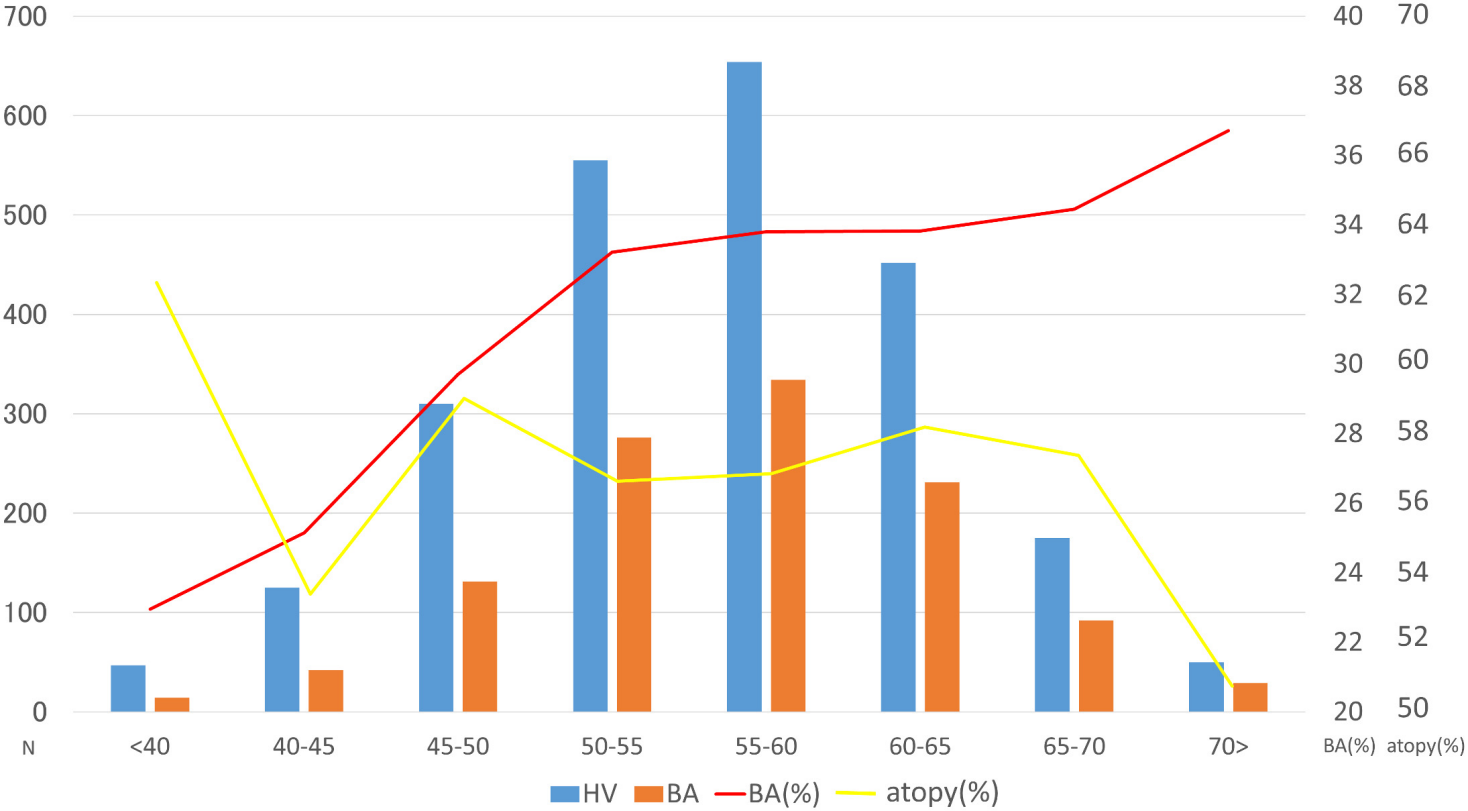
Prevalence of asthma according to GRS levels. The X-axis shows the GRS ranges. The left Y-axis shows the number of healthy individuals and asthmatic patients for each GRS range. The right Y-axis shows the percentages of asthmatic patients and atopic individuals for each GRS range. The upper line shows the percentage of atopic individuals for each given GRS range. The lower line shows the percentage of asthmatic patients for each given GRS range. Atopy was defined as the presence of specific IgE antibody toward at least 1 common inhaled allergen. HV, healthy volunteer, BA, bronchial asthma.

The Cochran-Armitage trend test showed that the prevalence of asthma increased as GRS levels increased (*P* = 0.0059) (Fig 1). In contrast, the prevalence of atopic status did not change according to GRS levels (*P* = 0.46), indicating that association between the GRS and presence of asthma was independent of atopic status.

In the sensitivity analysis, we calculated a GRS based on 15 SNPs excluding each of the 16 SNPs (S4 Table). Every GRS calculated based on each of the 15 SNPs was also significantly associated with asthma, suggesting that none of the single SNPs drives the significant association between a GRS and asthma in the current study.

**Fig 2:**
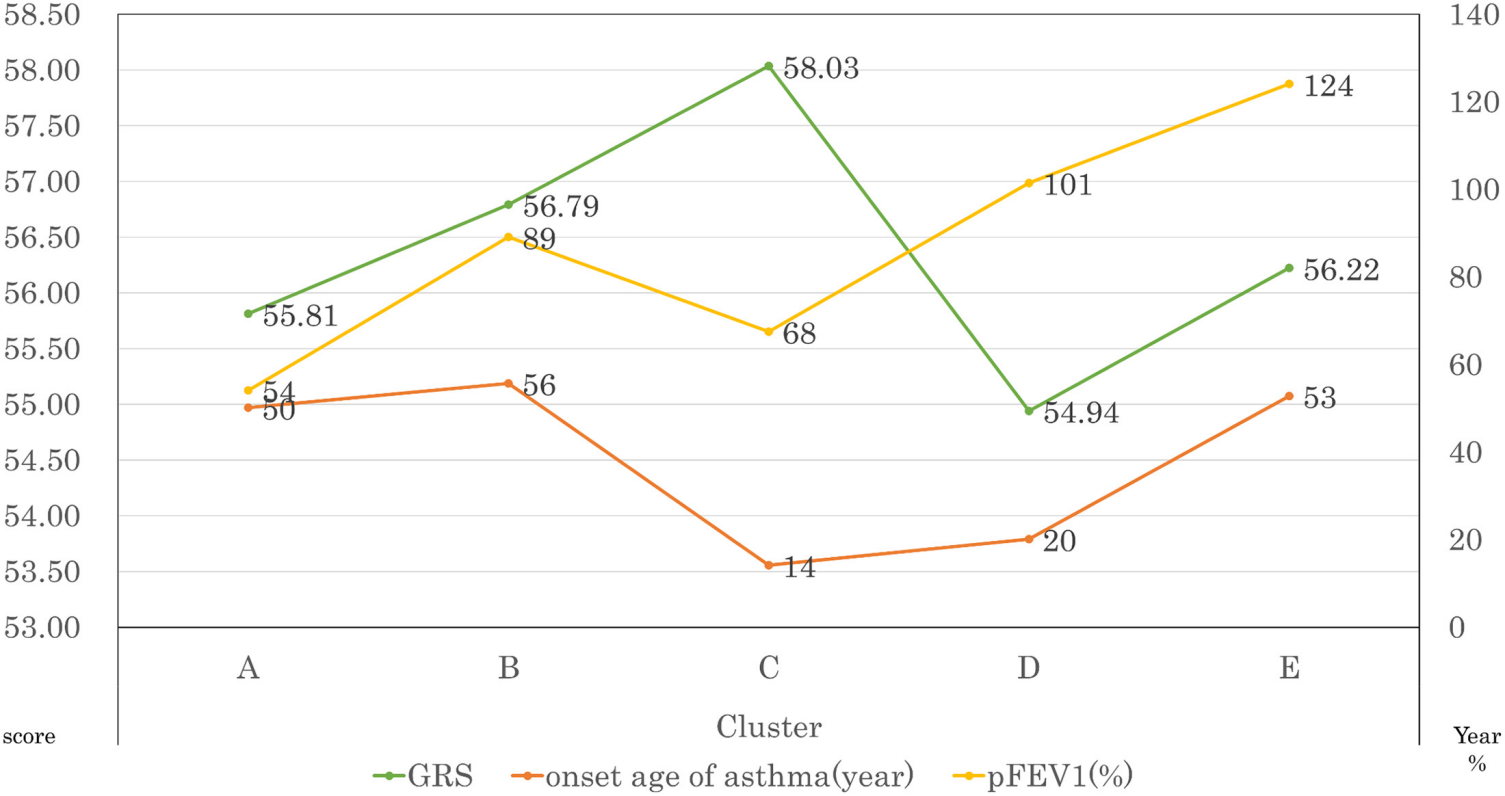
Differences in GRS, age at onset, and pFEV_1_ among asthma clusters. The left Y-axis shows the mean GRS for each cluster. The right Y-axis shows the mean age at onset asthma and the mean pFEV_1_ for each cluster. pFEV_1_, percent predicted forced expiratory volume in 1 second.

Our cluster analysis revealed 5 distinct asthmatic clusters (Fig 2). The cluster with the highest GRS (n = 197) was characterized by the youngest age at onset, moderate-to-severe lung function, the highest levels of total IgE, the highest prevalence of atopy (sensitizations to common inhaled allergens), and the lowest prevalence of smokers (S5 Table). In a multinomial logistic regression analysis, the strongest association between GRS levels and asthma was found in this particular cluster (*P* = 3.6×10^−5^, S6 Table).

**Fig 3:**
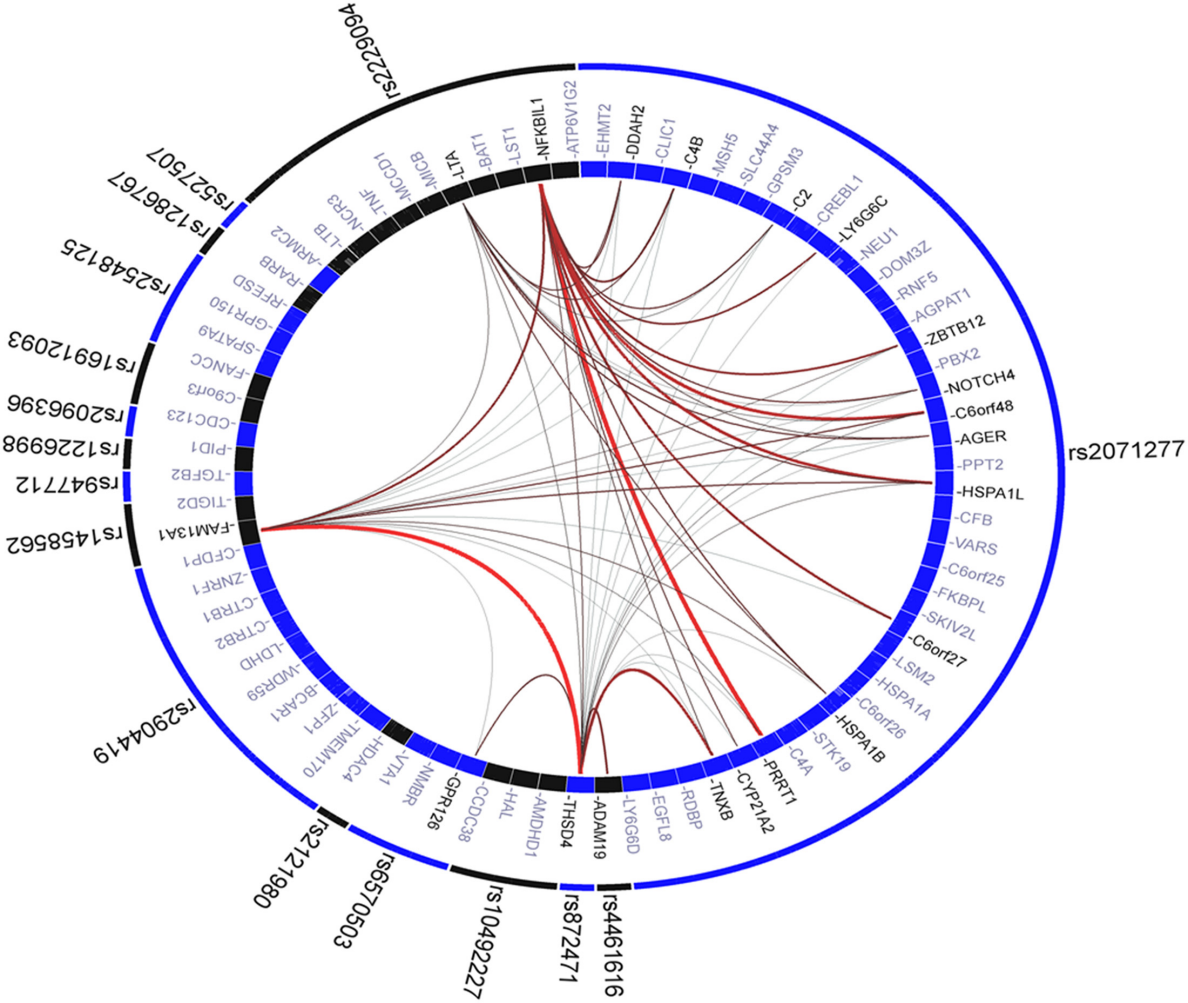
Graphic representation of functional connections between genes corresponding to the 16 SNPs. GRAIL found connections among genes in 16 loci. The outer circle shows the 16 SNPs used for the calculation of GRS. The internal ring represents the genes near each SNP. The literature-based functional connectivity between these genes with lines drawn between them—the redder and thicker the line is, the stronger the connectivity is between the genes.

To explore the biologic relevance of a GRS, we applied the top SNPs at 16 genes to the GRAIL program (Fig 3). For each SNP, GRAIL identified the genomic region in linkage disequilibrium and identified the overlapping genes. It then checked to see how many other loci already known to be associated with FEV_1_/FVC contained functionally related genes. GRAIL determined the corresponding probability values for all gene–gene connections. The outer ring contains the input SNPs, and the inner ring shows all possible genes corresponding to each SNP locus. GRAIL indicated the presence of possible underlying biologic networks across these 16 loci. *FAM13A*, one of the genes annotated by the 16 SNPs, was the most functionally connected gene when that locus was scored against the 15 other validated risk loci (S7 Table). MetaCore^™^ revealed several pathways associated with FEV_1_/FVC using the results of our GWAS (S8 Table). Among the top 20 pathways, two, “Signal transduction calcium signaling” and “Development regulation of epithelial-to-mesenchymal transition (EMT),” included genes that were annotated by GRAIL based on the 16 SNPs. Specifically, the EMT pathway contained 3 genes: *TGFB2*, *TNF*, and *NOTCH4*.

## Discussion

The GRS level for lower FEV_1_/FVC calculated based on the 16 SNPs was significantly higher in patients with asthma than in healthy adults, suggesting that a genetic susceptibility to lower lung function may predispose to asthma. Although both reduced growth and accelerated lung function decline lead to lower lung function levels in adults, given that the GRS calculated using 16 SNPs was not associated with annual lung function decline in the healthy participants, we believe that these 16 SNPs (or gene pathways involving these 16 SNPs) may be involved in the deregulated lung growth or development rather than in the accelerated decline of lung function. In support of this contention, lung function genetic variants identified in adults including *ARMC2*, *FAM13A*, *GPR126*, *KCNE2*, *NCR3*, *PID1*, *RARB*, and *THSD4* were recently associated with the development of lung function measures such as FEF50 and PD20 during early childhood [23].

We found that a GRS was associated with COPD as well as with asthma. The present study, therefore, may identify a common susceptibility factor underlying asthma and COPD, stressing the importance and implications of these genetic variants in the development of lung function in early life and later progression to asthma and COPD [24]. Accordingly, our findings support the Dutch hypothesis that airway obstruction associated with asthma and COPD is related to different expressions of a primary abnormality in the airways and that both host and environmental factors play a part in the pathogenesis [25]. Alternatively, our findings suggest that the asthma–COPD overlap can have its origins in childhood [26]

The difference in the levels of GRS between healthy individuals and asthmatic patient was rather small, which suggested that contribution of genetic susceptibility to lower lung function to asthma is restricted to particular subphenotypes of asthma. Indeed, our clustering of asthma patients according to GRS levels showed that the GRS was specifically related to a particular phenotype characterized by early-onset of the disease, atopy, and severer airflow obstruction, suggesting that deregulated lung function development ascribed to a GRS might be an important genetic determinant specific to this particular phenotype. In the Severe Asthma Research Program cohort, a similar cluster was identified that was characterized by early-onset atopic asthma with advanced airflow limitation. The prevalence of this cluster was 18%, and the children in this cluster had the highest exhaled nitric oxide values, the highest extent of health-care use, and the highest daily doses of ICS, and most were receiving at least 3 asthma controller medications [27]. The ‘‘Environment and Childhood Asthma” birth cohort study in Oslo also identified a similar asthma phenotype characterized by impaired lung function from birth throughout childhood with atopic dermatitis and allergic rhinitis [28]. Our clustering allowed greater specificity for a GRS as a genetic marker to identify this particular asthma phenotype/endotype related to deregulated lung function as well as better understanding of the mechanisms associated with the heterogeneity of severe asthma.

Our pathway analysis using GRAIL and MetaCore^™^ showed that molecules such as *TGFB2*, *TNF*, and *NOTCH4* that are related to the epithelial-mesenchymal transition (EMT) pathway could be responsible for the biologic relevance of a GRS. The EMT is a process whereby epithelial cells gradually transform into mesenchyme-like cells losing their epithelial functionality and characteristics. EMT is a physiologic phenomenon involved in lung development [29] and is also implicated in aberrant repair of the epithelium and accumulation of fibroblasts (tissue remodeling) in specific chronic bronchial pathologies, such as asthma and COPD [26]. Accordingly, together with our pathway analysis, the biologic relevance of a GRS, which was associated with both lung function and asthma or COPD, may involve the EMT process in the lung.

There are some limitations to this study. Even though the 16 genes used to create a GRS were selected on the basis of the previous GWASs conducted in non-Japanese populations, all the study participants were Japanese, and therefore the analysis provides limited information on other ethnic groups and may not reflect the overall asthma population. In addition, although the clustering of asthma patients using a GRS gave some implication underlying the heterogeneity of asthma, the likelihood always exists of spurious findings being generated by chance. Thus, further replication should aim to confirm these results, particularly given that the levels of significance in the study were not overwhelming.

In conclusion, we have identified a multi-SNP genetic marker, a GRS based on 16 SNPs, associated with a specific asthma phenotype related to genetic susceptibility to lower lung function. By providing important insights into the pathways underlying the development of asthma, the findings of this study may help us to understand the complexity of asthma phenotypes and also to appropriately select patients for more targeted prevention and treatments in the future.

## Supporting Information

S1 Fig Histogram of genetic risk scores (GRS) in healthy Japanese individuals. GRS in nonasthmatic, non-COPD healthy individuals of the Tsukuba cohort (N = 1364). The solid line indicates normal distribution.
(TIF)

S2 Fig Correlation between genetic risk score and FEV_1_/FVC or annual decline in FEV_1_ in healthy individuals. FEV_1_/FVC (a) or annual decline in FEV_1_ (b). Linear approximation is shown by the fine black line (a).
(TIF)

S3 Fig Differences in the GRS among the given asthma clusters according to the number of clusters. When *k*-means cluster analysis was repeated for the number of clusters (*k*) 3, 4, 5, 6, or 7, the maximum difference in the GRS among the *k* asthma clusters was obtained for *k* = 5 (ANOVA *F* = 4.8, *P* < 0.001).
(TIF)

S1 Table Demographic characteristics of the study cohorts. (DOCX)

S2 Table Susceptibility genes to pulmonary function traits in previous 6 GWASs. (DOCX)

S3 Table Sixteen genes associated with FEV_1_/FVC in previous GWASs are nominally replicated in Japanese. (DOCX)

S4 Table Association between GRS (by 15 SNPs) and asthma. (DOCX)

S5 Table Characteristics of the asthma clusters. (DOCX)

S6 Table Association between genetic risk score and asthma clusters. (DOCX)

S7 Table Functionally connected genes in GRAIL. (DOCX)

S8 Table Top 20 pathways implicated by MetaCore^™^. (DOCX)
