# Supplementary figures and images for: Role of Lung Function Genes in the Development of Asthma
Source: PLoS One. 2016 Jan 11;11(1):e0145832. doi: 10.1371/journal.pone.0145832 (PMC4709100; doi:10.1371/journal.pone.0145832)

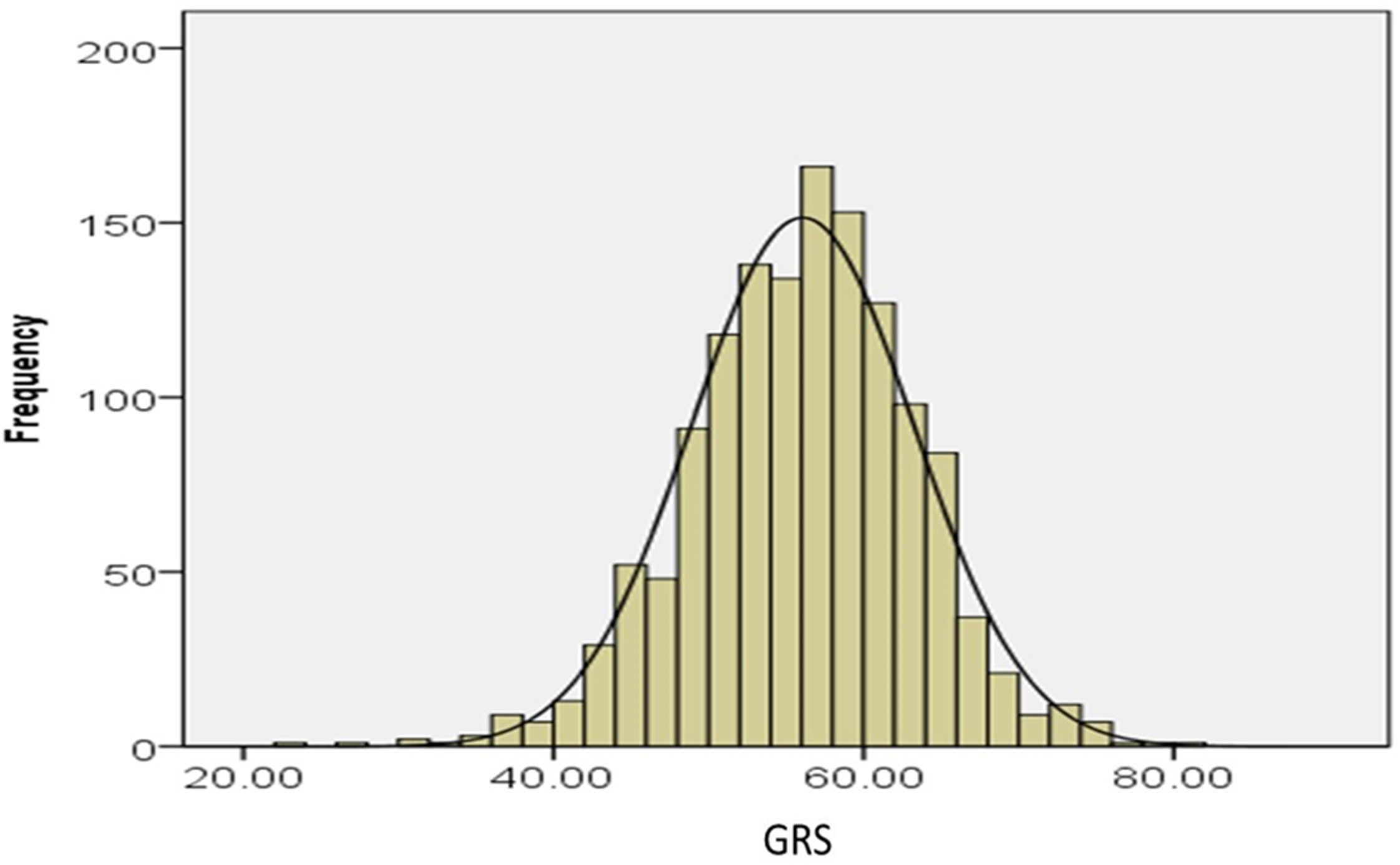

Supplement: S1 Fig — GRS in nonasthmatic, non-COPD healthy individuals of the Tsukuba cohort (N = 1364). The solid line indicates normal distribution. (TIF) [file pone.0145832.s001.TIF]

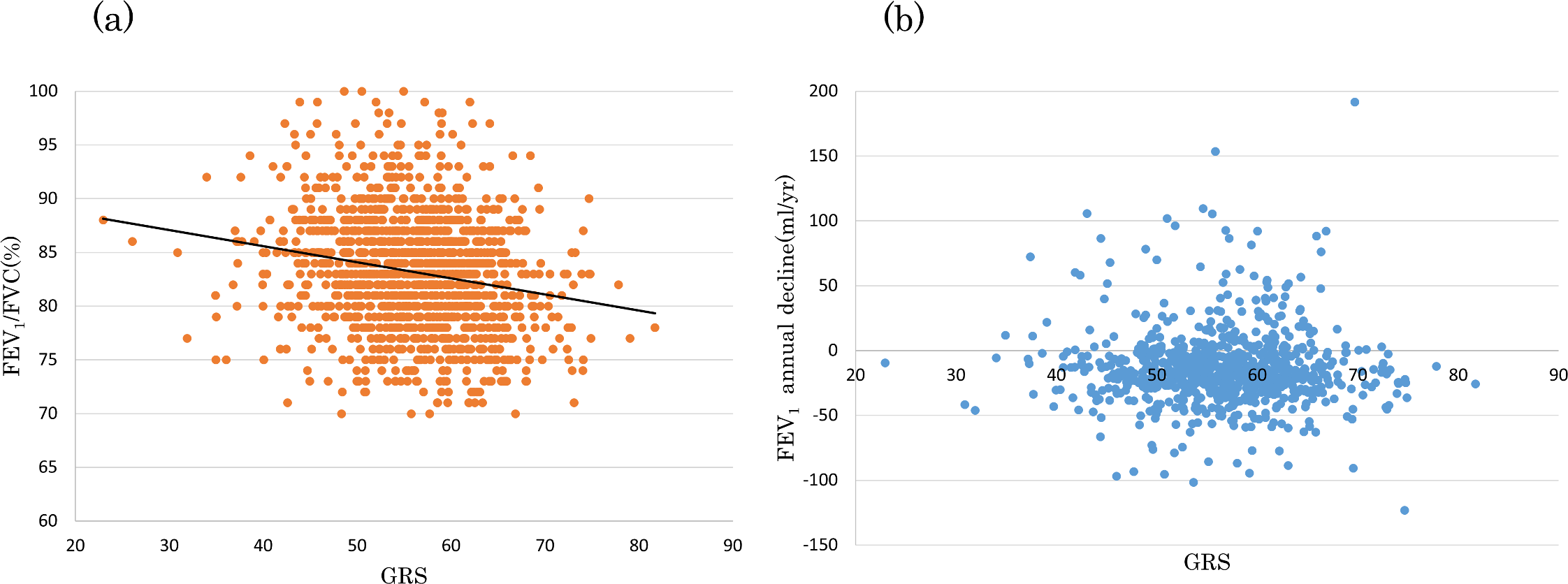

Supplement: S2 Fig — FEV1/FVC (a) or annual decline in FEV1 (b). Linear approximation is shown by the fine black line (a). (TIF) [file pone.0145832.s002.TIF]

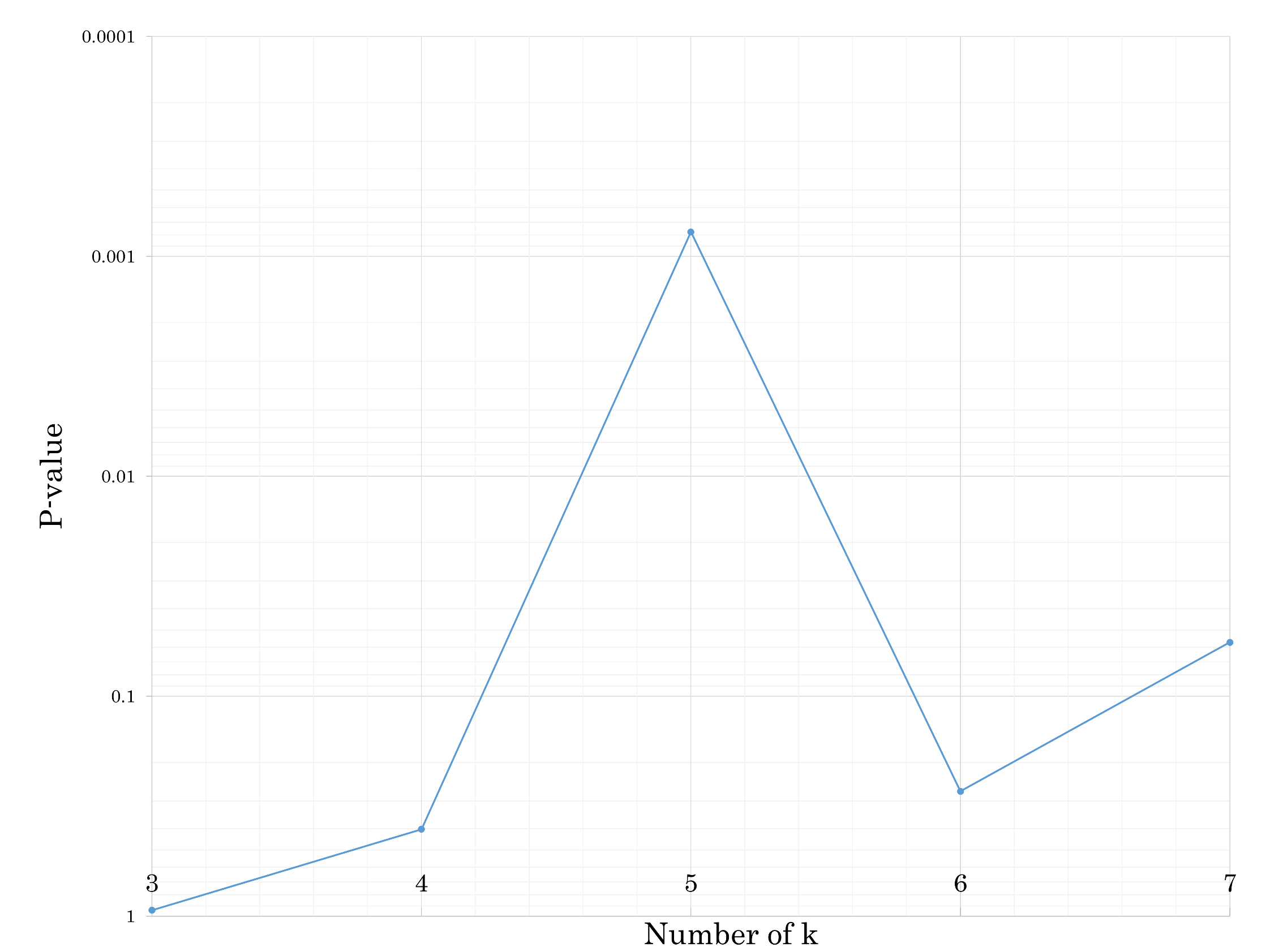

Supplement: S3 Fig — When k-means cluster analysis was repeated for the number of clusters (k) 3, 4, 5, 6, or 7, the maximum difference in the GRS among the k asthma clusters was obtained for k = 5 (ANOVA F = 4.8, P < 0.001). (TIF) [file pone.0145832.s003.TIF]
