# Supplementary material for: Role of Lung Function Genes in the Development of Asthma
Source: PLoS One. 2016 Jan 11;11(1):e0145832. doi: 10.1371/journal.pone.0145832 (PMC4709100; doi:10.1371/journal.pone.0145832)
Supplement: S1 Table — (DOCX) [file pone.0145832.s004.docx]

**S1 Table. Demographic characteristics of the study cohorts**

|  | | Tsukuba cohort | | Hokkaido cohort | | COPD cohort |
| --- | --- | --- | --- | --- | --- | --- |
|  |  | **HV (n = 1364)** | **BA (n = 578)** | **HV (n = 998)** | **BA (n = 565)** | **(n = 562)** |
| Age, y* | | 50.4 ± 9.6 | 56.9 ± 16.6 | 45.4 ± 13.5 | 52.2 ± 16.7 | 72.2 ± 8.8 |
| Female sex, n (%) | | 734 (53.8%) | 348 (60.2％) | 443 (44.4%) | 317 (56.2%) | 47 (8.4%) |
| Smoking index group  n(%) | 1 (0) | 852 (62.5%) | 390 (68.8%) | 530 (63.2%) | 296 (57.4%) | 10 (1.8%) |
|  | 2 (1-200) | 172 (12.6%) | 83 (14.6%) | 50 (6.0%) | 88 (17.1%) | 9 (1.6%) |
|  | 3 (> 200) | 340 (25.0%) | 94 (16.6%) | 258 (30.8%) | 132 (25.6%) | 542 (96.6%) |
| Atopic, n (%) | | 786 (57.6%) | 316 (71.2%) | 515 (51.8%) | 386 (69.3%) | N/A |
| Log total IgE* | | 1.78 ± 0.58 | 2.20 ± 0.62 | 1.83 ± 0.61 | 2.29 ± 0.66 | N/A |
| predicted FEV_1_, % | | 92.0% | 85.5% | 105.2% | 90.0% | 58.5% |
| FEV_1_/FVC, % | | 83.2% | 71.3％ | 83.0% | 68.1% | 48.7% |

FEV_1_, forced expiratory volume in the first second

FVC, forced vital capacity

HV, healthy volunteer

BA, bronchial asthma

COPD, chronic obstructive pulmonary disease

*Values are means ± SD
