# Supplementary material for: Role of Lung Function Genes in the Development of Asthma
Source: PLoS One. 2016 Jan 11;11(1):e0145832. doi: 10.1371/journal.pone.0145832 (PMC4709100; doi:10.1371/journal.pone.0145832)
Supplement: S2 Table — (DOCX) [file pone.0145832.s005.docx]

**S2 Table. Susceptibility genes to pulmonary function traits in previous 6 GWASs**

| **Pulmonary Function** | **Gene** | **Locus** | **Reference** |
| --- | --- | --- | --- |
| **FEV_1_** | *AGER* | 6p21.32 | [8, 9] |
| **FEV_1_** | *C10orf11* | 10q22.3 | [10] |
| **FEV_1_** | *GSTCD* | 4q24 | [8, 9] |
| **FEV_1_** | *HTR4* | 5q31-33 | [9] |
| **FEV_1_** | *MECOM* | 3q26 | [10] |
| **FEV_1_** | *THSD4* | 15q23 | [9] |
| **FEV_1_** | *TNS1* | 2q35-q36 | [9] |
| **FEV_1_** | *ZNF323* | 6p21.31 | [10] |
| **FEV_1_,FEV_1_/FVC** | *AGPHD1* | 15q25.1 | [12] |
| **FEV_1_,FEV_1_/FVC** | *CDC123* | 10p13 | [10] |
| **FEV_1_,FEV_1_/FVC** | *CHRNA3* | 15q24 | [12] |
| **FEV_1_,FEV_1_/FVC** | *CHRNA5* | 15q24 | [12] |
| **FEV_1_,FEV_1_/FVC** | *HHIP* | 4q28-32 | [8, 9, 11] |
| **FEV_1_/FVC** | *ADAM19* | 5q33.3 | [8] |
| **FEV_1_/FVC** | *ARMC2* | 6q21 | [10] |
| **FEV_1_/FVC** | *CCDC38* | 12q23.1 | [10] |
| **FEV_1_/FVC** | *CFDP1* | 16q22.2 | [10] |
| **FEV_1_/FVC** | *FAM13A* | 4q22.1 | [8] |
| **FEV_1_/FVC** | *GPR126* | 6q24.1 | [8] |
| **FEV_1_/FVC** | *HDAC4* | 2q37.3 | [10] |
| **FEV_1_/FVC** | *KCNE2* | 21q22.12 | [10] |
| **FEV_1_/FVC** | *LRP1* | 12q13-q14 | [10] |
| **FEV_1_/FVC** | *MFAP2* | 1p36 | [10] |
| **FEV_1_/FVC** | *MMP15* | 16q13 | [10] |
| **FEV_1_/FVC** | *NCR3* | 6p21.3 | [10] |
| **FEV_1_/FVC** | *PID1* | 2q36.3 | [8] |
| **FEV_1_/FVC** | *PPT2* | 6q21.3 | [8] |
| **FEV_1_/FVC** | *PTCH1* | 9q22.3 | [8] |
| **FEV_1_/FVC** | *RARB* | 3p24.2 | [10] |
| **FEV_1_/FVC** | *SPATA9* | 5q15 | [10] |
| **FEV_1_/FVC** | *TGFB2* | 1q41 | [10] |
| **FEV_1_/FVC** | *THSD4* | 15q23 | [13] |

In previous GWASs, 24 genes showed associations with FEV1/FVC at the level of genomewide significance.
