# Supplementary material for: Role of Lung Function Genes in the Development of Asthma
Source: PLoS One. 2016 Jan 11;11(1):e0145832. doi: 10.1371/journal.pone.0145832 (PMC4709100; doi:10.1371/journal.pone.0145832)
Supplement: S3 Table — (DOCX) [file pone.0145832.s006.docx]

**S3 Table. Sixteen genes associated with FEV_1_/FVC in previous GWASs are nominally replicated in Japanese**

| **genes** | **Top SNP** | **Risk allele** | **P** |
| --- | --- | --- | --- |
| ***ADAM19**** | rs4461616 | G | 0.0493 |
| ***AGPHD1*** | rs12441998 | G | 0.0931 |
| ***ARMC2**** | rs527507 | A | 0.0147 |
| ***CCDC38**** | rs10492227 | C | 0.0112 |
| ***CDC123**** | rs2096396 | A | 0.01622 |
| ***CFDP1**** | rs2904419 | C | 0.0391 |
| ***CHRNA3*** | rs12441998 | G | 0.0931 |
| ***CHRNA5*** | rs12441998 | G | 0.0931 |
| ***FAM13A**** | rs1458562 | A | 9.42 x 10^-4^ |
| ***GPR126**** | rs6570503 | C | 0.049 |
| ***HDAC4**** | rs2121980 | T | 0.0142 |
| ***HHIP*** | rs6537307 | G | 0.13 |
| ***KCNE2*** | rs2834484 | G | 0.0648 |
| ***LRP1*** | rs11172113 | T | 0.0835 |
| ***MFAP2*** | rs3754511 | T | 0.105 |
| ***MMP15*** | rs9931166 | G | 0.0588 |
| ***NCR3**** | rs2229094 | C | 4.89 x 10^-4^ |
| ***PID1**** | rs1226998 | T | 0.0201 |
| ***PPT2**** | rs2071277 | G | 5.93 x 10^-3^ |
| ***PTCH1**** | rs16912093 | C | 3.45 x 10^-3^ |
| ***RARB**** | rs1286767 | A | 0.022 |
| ***SPATA9**** | rs2548125 | C | 0.0236 |
| ***TGFB2**** | rs947712 | A | 0.0262 |
| ***THSD4**** | rs872471 | G | 2.46 x 10^-3^ |

SNP, single nucleotide polymorphism

**P* value < 0.05
