# Supplementary material for: Role of Lung Function Genes in the Development of Asthma
Source: PLoS One. 2016 Jan 11;11(1):e0145832. doi: 10.1371/journal.pone.0145832 (PMC4709100; doi:10.1371/journal.pone.0145832)
Supplement: S4 Table — (DOCX) [file pone.0145832.s007.docx]

**S4 Table. Association between GRS (by 15 SNPs) and asthma**

| **Excluded SNP** | ***P* value** |
| --- | --- |
| **rs4461616** | 5.6 × 10^-5^ |
| **rs527507** | 0.0010 |
| **rs10492227** | 8.1 × 10^-4^ |
| **rs2096396** | 0.0030 |
| **rs2904419** | 9.0 × 10^-5^ |
| **rs1458562** | 2.0 × 10^-5^ |
| **rs6570503** | 7.0 × 10^-4^ |
| **rs2121980** | 0.0017 |
| **rs2229094** | 0.047 |
| **rs1226998** | 0.0029 |
| **rs2071277** | 0.026 |
| **rs16912093** | 0.0025 |
| **rs1286767** | 5.2 × 10^-4^ |
| **rs2548125** | 0.0048 |
| **rs947712** | 3.6 × 10^-4^ |
| **rs872471** | 0.0048 |
