# Supplementary material for: Role of Lung Function Genes in the Development of Asthma
Source: PLoS One. 2016 Jan 11;11(1):e0145832. doi: 10.1371/journal.pone.0145832 (PMC4709100; doi:10.1371/journal.pone.0145832)
Supplement: S5 Table — (DOCX) [file pone.0145832.s008.docx]

**S5 Table. Characteristics of the asthma clusters**

| **Cluster** | **A**  **n = 167** | **B**  **n = 318** | **C**  **n = 197** | **D**  **n = 220** | **E**  **n = 161** | ***P* value** |
| --- | --- | --- | --- | --- | --- | --- |
| **GRS** | 55.81 | 56.79 | 58.03 | 54.94 | 56.22 | < 0.001 |
| **Age** | 62 | 64 | 43 | 43 | 62 | < 0.001 |
| **Age at onset** | 50 | 56 | 14 | 20 | 53 | < 0.001 |
| **pFEV_1_, %** | 54 | 89 | 68 | 101 | 124 | < 0.001 |
| **FEV_1_/FVC, %** | 57 | 69 | 66 | 78 | 77 | < 0.001 |
| **Log IgE, IU/L** | 2.3 | 2.2 | 2.4 | 2.3 | 2 | < 0.001 |
| **Body mass index** | 23 | 24 | 24 | 24 | 23 | 0.769 |
| **Sex, female %** | 56 | 64 | 45 | 65 | 61 | < 0.001 |
| **Atopy, %** | 56 | 46 | 75 | 74 | 58 | < 0.001 |
| **Never-smoker, %** | 54 | 64 | 73 | 64 | 58 | < 0.001 |

Cluster C, which had the highest GRS, was characterized by the youngest age at onset, moderate-to-severe lung function, the highest levels of total IgE, the highest sensitizations to common inhaled allergens (atopy), and the lowest prevalence of smokers.

GRS, genetic risk score for lower FEV_1_/FVC

pFEV_1_, percent predicted forced expiratory volume in 1 second
