# Supplementary material for: Role of Lung Function Genes in the Development of Asthma
Source: PLoS One. 2016 Jan 11;11(1):e0145832. doi: 10.1371/journal.pone.0145832 (PMC4709100; doi:10.1371/journal.pone.0145832)
Supplement: S6 Table — (DOCX) [file pone.0145832.s009.docx]

**S6 Table. Association between genetic risk score and asthma clusters**

| Cluster | A | B | C | D | E |
| --- | --- | --- | --- | --- | --- |
| Odds ratio  (95% CI) | 0.98  [0.90-1.07] | 1.09  [1.01-1.15] | 1.19  [1.09-1.26] | 0.96  [0.92-1.04] | 1.10  [1.00-1.19] |
| *P* | 0.71 | 0.016 | 8.4×10^-5^ | 0.33 | 0.037 |
| *P* (adjusted)* | 0.74 | 0.016 | 3.6×10^-5^ | 0.62 | 0.040 |

CI, confidence interval

To examine an association of a GRS with each of the 5 asthma clusters in comparison with healthy volunteers (n = 2362), multinomial logistic regression was modelled.

*Adjusted for sex, age, atopy, smoking index group, and cohorts
