# Supplementary material for: Role of Lung Function Genes in the Development of Asthma
Source: PLoS One. 2016 Jan 11;11(1):e0145832. doi: 10.1371/journal.pone.0145832 (PMC4709100; doi:10.1371/journal.pone.0145832)
Supplement: S7 Table — (DOCX) [file pone.0145832.s010.docx]

**S7 Table. Functionally connected genes in GRAIL**

| **Gene** | **GRAIL *P* value** |
| --- | --- |
| *FAM13A1* | 0.00083 |
| *HSPA1L* | 0.0042 |
| *C6orf48* | 0.0050 |
| *AGER* | 0.0056 |
| *TNXB* | 0.0059 |
| *PRRT1* | 0.0069 |
| *NFKBIL1* | 0.0087 |
| *GPR126* | 0.011 |
| *C6orf27* | 0.016 |
| *DDAH2* | 0.020 |
| *HSPA1B* | 0.024 |
| *ADAM19* | 0.024 |
| *LTA* | 0.029 |
| *THSD4* | 0.031 |
| *ZBTB12* | 0.034 |
| *C2* | 0.034 |
| *NOTCH4* | 0.035 |
| *CYP21A2* | 0.041 |
| *LY6G6C* | 0.043 |
| *C4B* | 0.044 |
