# Supplementary material for: Role of Lung Function Genes in the Development of Asthma
Source: PLoS One. 2016 Jan 11;11(1):e0145832. doi: 10.1371/journal.pone.0145832 (PMC4709100; doi:10.1371/journal.pone.0145832)
Supplement: S8 Table — (DOCX) [file pone.0145832.s011.docx]

**S8 Table. Top 20 pathways implicated by MetaCore^TM^**

| **#** | **Maps** | **Total number of genes** | ***P* value** | **Min FDR** | **In Data** |
| --- | --- | --- | --- | --- | --- |
| **1** | Cell adhesion_Ephrin signaling | 45 | 8.9 × 10^-7^ | 3.7 × 10^-4^ | 17 |
| **2** | Development_c-Kit ligand signaling pathway during hemopoiesis | 61 | 1.4 × 10^-6^ | 3.7 × 10^-4^ | 20 |
| **3** | Cytoskeleton remodeling_TGF, WNT and cytoskeletal remodeling | 111 | 1.4 × 10^-6^ | 3.7 × 10^-4^ | 29 |
| **4** | Signal transduction_Activation of PKC via G-Protein coupled receptor | 52 | 1.9 × 10^-6^ | 3.7 × 10^-4^ | 18 |
| **5** | Signal transduction_Calcium signaling | 45 | 4.7 × 10^-6^ | 7.1 × 10^-4^ | 16 |
| **6** | Cell adhesion_Chemokines and adhesion | 100 | 5.5 × 10^-6^ | 7.1 × 10^-4^ | 26 |
| **7** | Signal transduction_ERK1/2 signaling pathway | 32 | 6.8 × 10^-6^ | 7.6 × 10^-4^ | 13 |
| **8** | Cytoskeleton remodeling_Cytoskeleton remodeling | 102 | 8.1 × 10^-6^ | 7.8 × 10^-4^ | 26 |
| **9** | Development_WNT5A signaling | 47 | 9.0 × 10^-6^ | 7.8 × 10^-4^ | 16 |
| **10** | G-protein signaling_RhoA regulation pathway | 34 | 1.5 × 10^-5^ | 1.2 × 10^-3^ | 13 |
| **11** | Neurophysiological process_Long-term depression in cerebellum | 49 | 1.7 × 10^-5^ | 1.2 × 10^-3^ | 16 |
| **12** | Translation_Non-genomic (rapid) action of Androgen Receptor | 40 | 2.3 × 10^-5^ | 1.4 × 10^-3^ | 14 |
| **13** | Development_Thrombopoietin-regulated cell processes | 45 | 2.3 × 10^-5^ | 1.4 × 10^-3^ | 15 |
| **14** | Neurophysiological process_Constitutive and regulated NMDA receptor trafficking | 62 | 3.1 × 10^-5^ | 1.6 × 10^-3^ | 18 |
| **15** | Main growth factor signaling cascades in multiple myeloma cells | 41 | 3.2 × 10^-5^ | 1.6 × 10^-3^ | 14 |
| **16** | Neurophysiological process_NMDA-dependent postsynaptic long-term potentiation in CA1 hippocampal neurons | 80 | 3.7 × 10^-5^ | 1.8 × 10^-3^ | 21 |
| **17** | Development_WNT signaling pathway. Part 1. Degradation of beta-catenin in the absence WNT signaling | 19 | 4.3 × 10^-5^ | 2.0 × 10^-3^ | 9 |
| **18** | Development_Regulation of epithelial-to-mesenchymal transition (EMT) | 64 | 4.9 × 10^-5^ | 2.1 × 10^-3^ | 18 |
| **19** | Signal transduction_cAMP signaling | 38 | 5.9 × 10^-5^ | 2.3 × 10^-3^ | 13 |
| **20** | Development_Gastrin in differentiation of the gastric mucosa | 38 | 5.9 × 10^-5^ | 2.3 × 10^-3^ | 13 |

FDR, false discovery rate
